# Percutaneous-Reinforced Osteoplasty: A Review of Emerging Treatment Strategies for Bone Interventions

**DOI:** 10.3390/jcm11195572

**Published:** 2022-09-22

**Authors:** Nischal Koirala, Jyotsna Joshi, Stephen F. Duffy, Gordon McLennan

**Affiliations:** 1 Department of Chemical and Biomedical Engineering, Cleveland State University, Cleveland, OH 44115, USA; 2 Department of Biomedical Engineering, Cleveland Clinic, Cleveland, OH 44195, USA; 3 Department of Civil and Environmental Engineering, Cleveland State University, Cleveland, OH 44115, USA

**Keywords:** bone biomechanics, cementoplasty, mechanical testing, percutaneous osteoplasty, three-point/four-point flexural test

## Abstract

Percutaneous-reinforced osteoplasty is currently being investigated as a possible therapeutic procedure for fracture stabilization in high-risk patients, primarily in patients with bone metastases or osteoporosis. For these patients, a percutaneous approach, if structurally sound, can provide a viable method for treating bone fractures without the physiologic stress of anesthesia and open surgery. However, the low strength of fixation is a common limitation that requires further refinement in scaffold design and selection of materials, and may potentially benefit from tissue-engineering-based regenerative approaches. Scaffolds that have tissue regenerative properties and low inflammatory response promote rapid healing at the fracture site and are ideal for percutaneous applications. On the other hand, preclinical mechanical tests of fracture-repaired specimens provide key information on restoration strength and long-term stability and enable further design optimization. This review presents an overview of percutaneous-reinforced osteoplasty, emerging treatment strategies for bone repair, and basic concepts of in vitro mechanical characterization.

## 1. Introduction

Bone disease and traumatic fractures are the most common orthopedic problems worldwide with huge societal and economic effects [1,2]. Osteoporosis and bone metastasis are the most common bone diseases responsible for compromised bone strength predisposing individuals to increased risks of fractures [3,4]. According to a recent study, in the United States alone, approximately 350,000 people are estimated to die each year from bone metastasis with the highest incidences among people with metastatic disease of the prostate, breast, and kidney [5]. Fractures occurring in patients with bone metastasis or osteoporosis are difficult to repair as the bone strength and the overall health are severely compromised prohibiting invasive and/or time-consuming surgical procedures. Often these patients have progressed disease and are unable to perform their daily activities independently and live with pain, reduced quality of life, and poor prognosis. For these patients, a percutaneous approach, if structurally sound, can provide a viable method for treating fractures without the physiologic stress of anesthesia and open surgery, and positively impact the quality of their remaining life. However, low fracture strength is a common drawback that requires further research in scaffold design and development, including in vitro and in vivo biomechanical characterizations. Current clinical strategies for managing bone metastases in interventional radiology (IR) settings have been provided (Table 1). 

Mechanical testing data provide information about the performance of the device (maximum load, fracture/failure, etc.) under various (simulated) loading conditions, whereas, assays (histopathology, immunohistochemistry, microCT, etc.) involving biological specimens provide information regarding cell/tissue and device interactions, allowing researchers to predict possible complications when used in humans [6]. It is, therefore, necessary to have a good understanding of the various testing methods and parameters that can be used for relevant mechanical quantification of bone interventional devices. The structural support rendered by the osteoplasty technique and its variations is the cornerstone of pain relief and ambulation therapy making mechanical evaluation crucial to assess the benefits of these procedures. Mechanical characterization of the bone can be achieved at the macroscopic level or the micro/nano level depending on the size of the specimen used for testing [7,8]. At the macroscopic level, the whole bone is usually subjected to mechanical testing, which provides an assessment of the bone’s extrinsic parameters such as stiffness or load to failure; these factors are specific to the specimen subjected to the test. On the other hand, tests performed using small-sized specimens are targeted to determine the intrinsic material properties such as stress, strain, elasticity, and ultimate/breaking strength, and these properties are independent of the geometrical specifications of the specimen. In this review, we inform the progress and outlook of percutaneous bone interventional research, and briefly outline various testing methods available for in vitro mechanical characterization along with relevant physical parameters that are of particular interest.

**Table 1:** Currently available therapies for the management of bone metastases with IR.

IR technique.	Description	Advantages and Disadvantages	Ref.
Mechanical stabilization			
Cementoplasty	Use of bone cement for bone consolidation and pain palliation.	Widely available procedure at low cost. PMMA cement has low resistance to bending and twisting, toxicity, and cement leakage.	[9,10]
Reinforcement cementoplasty	Use of reinforcements such as K-wires, nails, and screws in addition to regular cementoplasty.	Provides additional mechanical stability. Infection, pain, and reinforcement failures.	[11,12,13,14]
Tumor destruction			
Ablation—Radiofrequency (RFA) and microwave (MWA)	Tumor destruction with the application of high temperature (≥60 ° C).	RFA—Cost-effective, widely used. Suffers from local perfusion or vascular heat sink effects, less powerful than MWA, and non-homogeneous energy propagation. MWA—Minimal heat sink effect, independent of tissue non-conductivity, and larger ablation zones. Imprecise energy delivery with oval ablation effects, and overheating.	[15,16]
Cryoablation	Tumoricidal effects from exposure to extremely cold temperatures of super-cooled gases (<−40 °C).	Deep tissue penetration, ablated areas visible in imaging due to ice-ball formation (temperature difference), and less damage to tissue architecture. Costlier than RFA or MWA, cryoshock, and not indicated for all tumors.	
Embolization	Obstruction of tumor-feeding blood vessels that cuts off nutrients and oxygen supply (devascularization) choking tumor cells.	Less bleeding and rapid cut-off of blood supply. Infection, ischemia, and potential damage to healthy tissues from wrong delivery.	[15,17]
MR-guided Focused Ultrasound (MRgFUS) (also called, High-Intensity Focused Ultrasound [HIFU]).	Application of focussed ultrasonic pulses to lyse tumor cells with MR-based tissue identification and targeting.	Minimally invasive and does not require tissue contact via needlelike applicators to deliver ultrasonic waves. Shallow penetration depth.	

## 2. Percutaneous-Reinforced Bone Interventions

**Figure 1:**
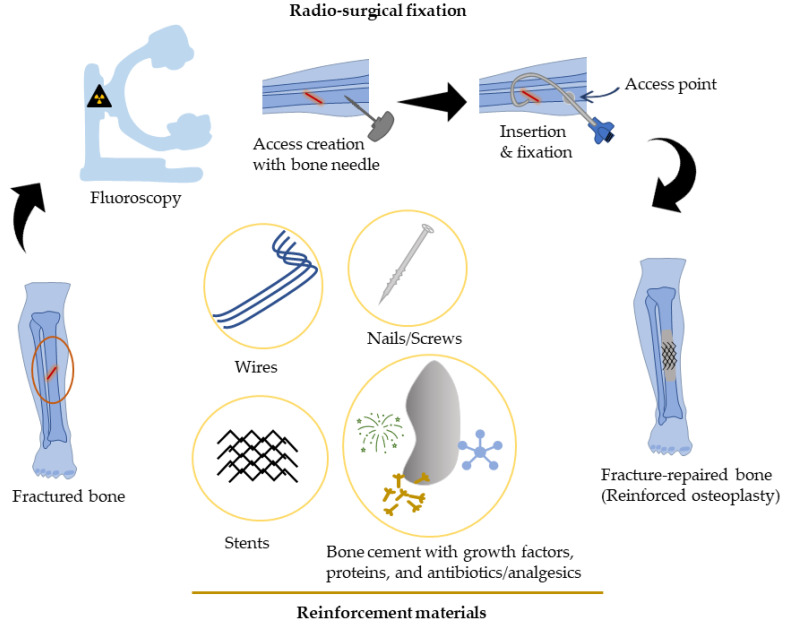
Block diagram illustrating percutaneous-reinforced osteoplasty procedure. A mid-diaphyseal fracture of the tibia in a strictly “non-surgical” patient is repaired radio-surgically by creating access with a bone biopsy needle followed by insertion of reinforcement material(s) and cement augmentation.

Percutaneous osteoplasty/cementoplasty (PC) is a minimally invasive technique common in radiology clinics for pain relief, bone consolidation, and stabilization of impending fractures of the (extraspinal) skeletal tissues [18]. Under fluoroscopic guidance, cement is injected via catheters and guidewires into the bone through a small hole (“access”) drilled at the skin surface [19,20,21,22,23]. A block diagram representing percutaneous osteoplasty with reinforcement has been illustrated (Figure 1). Multiple studies have verified the benefits of PC for pain management and stabilization of impending/pathological fractures in osteolytic malignant tumors [24,25,26]. During this procedure, the injected cement percolates into the bone cavity, filling the voids and closing the fracture clefts that provide structural stability, and this mechanism has been primarily associated with pain palliation [23]. Some studies have postulated that heat produced from the exothermic reaction of polymethylmethacrylate (PMMA) polymerization results in the local necrosis of painful nerves causing pain relief and tumor control [25,27,28]. However, the theory of pain relief connected with heat emanation and/or cement monomer toxicity has been contradicted in several studies. Anselmetti et al. used biological calcium-phosphate bone cement, which had no exothermic reaction upon cement hardening and toxicity, for the treatment of benign lesions of the bone and, nevertheless, achieved pain relief [23]. This supported the notion that bone matrix stabilization and prevention of microfracture from cement injection produce pain relief. Poussot et al. have pointed out the mechanical stabilization from the insertion of internal cement screws for sternal fracture fixation or consolidation of osteolytic metastasis promulgated pain relief [29]. The rise in temperature from PMMA cement polymerization is not standardized and varies widely (39 °C to >100 °C) depending on its composition and site of measurement [30]. In addition, not all areas of the bone uniformly receive cement distribution. Therefore, local necrosis and/or tumoricidal effects as a result of the thermal ablative effects from cement hardening cannot be assumed confidently [30]. In one study, PC performed on 20 patients with a history of painful lytic bone lesions led to a significant decrease in pain for a relatively longer duration (average follow-up duration, 7.75 months) [31]. Overall, 64% of patients treated for lower limb and pelvic lesions had improved mobility. In another study comprising 5 patients with a history of malignancy and metastases in the pelvis and femur, PC led to immediate pain relief without the need for any analgesics. Patients were discharged on the same day after the procedure and did not experience any clinically significant complications [32]. Similarly, microwave ablation and PC have been used for pain control and local tumor control of extraspinal osseous tumors. The study was conducted on 65 patients and in total 77 tumors were treated in multiple locations. Over 64% of patients showed no local progression (stable disease) on follow-up imaging at 20–24 weeks. The procedure resulted in pain relief, structural stability, and locoregional tumor control [33]. Similarly, Plancarte-Sanchez et al. have reported that femoroplasty (percutaneous cement injection in the femur) resulted in pain reduction and improvement in mobility without complications in 15 patients who had bone metastases located in the proximal femur [34]. In the subsequent larger study (*n* = 80), femoroplasty resulted in a decreased intensity of pain, reduced analgesic consumption, and improved quality of life, at 7 and 30 days after the intervention [35]. No serious complications were noted. In another study, radiofrequency ablation and percutaneous osteoplasty have been used for pain relief and functional recovery in patients with bone metastases [20]. The procedure resulted in an immediate reduction in pain in the majority of the patients (92.1%). The mean visual analog scale (VAS) score before treatment was 7.1 ± 1.5 versus 2.2 ± 2.0 at 24 h post-treatment, and continued to reduce substantially thereafter (1.6 ± 1.8 (at 3 months), 1.3 ± 1.8 (at 6 months)). The Karnofsky performance scale score was reported significantly higher after the treatment. These results attest that PC provides bone stability and pain relief in osseous-compromised patients within a relatively short recovery period.

Bone cement has been in regular use for vertebral compression and fracture management in vertebroplasty and kyphoplasty procedures. However, the fracture strength and flexibility of cement-only repaired bone are lower than that of an intact bone. Since long bones are subject to twisting and are more susceptible to fracture in torsion, cement injection alone may not add sufficient mechanical stability. Studies have shown supplemental reinforcement, e.g., cement-filled catheter [36] or percutaneous osteosynthesis [37], in conjunction with cementoplasty adds functional improvement and prevents impending pathological fracture in symptomatic patients with extraspinal malignant bone lesions [13,38,39]. Despite potential clinical applications, the advantages of reinforced osteoplasty remain a relatively under-explored area. Some commonly used materials for percutaneous osteoplasty along with their advantages and disadvantages have been briefly summarized (Table 2).

**Table 2:** Materials commonly used for reinforcements in percutaneous osteoplasty.

Material	Evidence	Advantages	Disadvantages	Ref.
PMMA bone cement	Preclinical Clinical	Readily available, ease of use, high axial compressive strength (80–94 MPa).	Low bending (67–72 MPa), tensile (36–47 MPa; Young’s modulus: ~2400 MPa), and shear strength (50–69 MPa).	[40]
Calcium phosphate cement	Clinical	Good osteoconductive properties, mimics natural mineral phase of bone, low toxicity.	More expensive than PMMA cement, and has low mechanical strength (compressive strength: 35 MPa).	[41,42]
Stents	Preclinical	Ease of deployment in the target site.	Low mechanical strength, and displacement.	[12,43,44]
Nails (Screws)	Preclinical Clinical	Bone consolidation, stability, and durability.	Breaking, loosening, or migration.	[45,46,47]
K-wires	Preclinical Clinical	Ease of deployment, deployable in bundles to boost strength.	Low mechanical strength.	[48,49]
Y STRUTS	Preclinical Clinical	Bone consolidation, reinforces mechanical stability.	Increase in procedural complexity and time, application limited to proximal femur.	[50,51]

Percutaneous fracture fixation and the development of scaffolds to reinforce bone strength are advanced concepts in fracture management and osseous stabilization [13,21,38,39,43,44,52]. In particular, percutaneous osteoplasty with acrylic bone cement only or in combination with other stiffer materials (reinforcement) that mimic internal fixators have been used to treat bone disease, leading to reduced pain, improved strength, and enhanced mobility [53,54]. Kawaii and colleagues have performed percutaneous osteoplasty using a cement-filled catheter and (acrylic) cement augmentation to reunite a painful pathological fracture of the humerus shaft in a patient with metastatic hepatocellular carcinoma [39]. The procedure was offered because of the patient’s deteriorated health and poor prognosis, which imposed a high risk for open reduction methods. The procedure resulted in immediate pain relief and improved limb mobility; however, the fixation strength was unsatisfactory because of the low durability of the cement-filled catheter. In another study, acrylic cement was used in a patient with lymphangiomatosis in long bones (left femur and tibia) to provide pain relief and structural support to the compromised bone [53]. After over 2 years of follow-up, the radiological findings showed good intramedullary support and no signs of active disease progression. Similarly, another study in patients with femoral metastasis found that percutaneous osteoplasty with internal fixators led to improvement in pain relief, increased bone consolidation, and a decrease in incidences of fracture when compared to percutaneous osteoplasty without fixators during a follow-up period of 3–18 months [54]. These studies provide testimony that percutaneous osteoplasty with reinforcement has potential benefits in terms of improved mobility, pain reduction, and overall enhancement in quality of life.

### Percutaneous Bone Intervention Procedure

**Figure 2:**
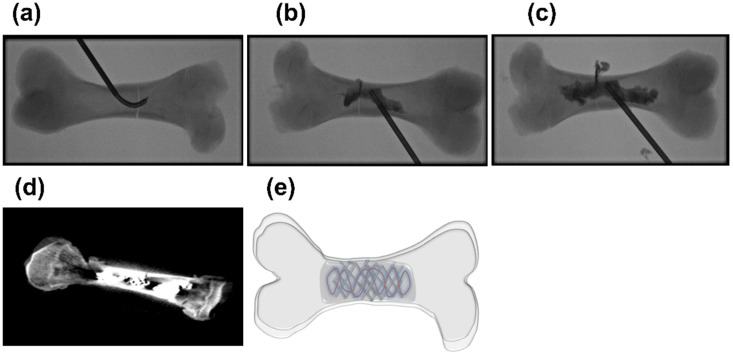
Percutaneous-reinforced osteoplasty procedure [12]. (**a**) Access to the intramedullary canal is established with a bone needle through a cortical hole adjacent to the fracture line, (**b**) cement injection (initial phase) via bone cannula, (**c**) cement injection (final phase), (**d**) stent and wire scaffolding in position following cementoplasty, (**e**) artistic representation of stent-wire-cement scaffolding strategy (percutaneous-reinforced osteoplasty). (Parts of figures were reprinted with permission, © 2020 Springer, [12]).

The scaffolds commonly used in percutaneous repair for proof-of-concept evaluation include bone cement and stents, which can be injected over the wire and safely placed in the region of interest. The procedure of fracture fixation is performed under fluoroscopic guidance. In this procedure, a guidewire is advanced into the bone cavity through access to the cortex (Figure 2). The stent is then deployed at the fracture site, and other reinforcement materials (if any) may be placed within the stent lumen to impart additional strength—mimicking a “rebar” concept of a construction setting. After securely depositing all repair materials in position, cement is injected through the catheter filling the intramedullary canal. Bonding with the injected materials is instantly achieved and results in a rigid scaffold able to undertake varying loads at the fractured site [44,52]. 

In one study, in vivo safety and feasibility of percutaneous fracture repair using a “bone marrow nail” have been evaluated in a swine model [44]. The nail was made in vivo through the insertion of a covered metallic stent in the intramedullary canal of the humerus and tibia, followed by cement augmentation; a corresponding in vitro nail was made of acrylic cement. Blood results and pathological findings in the swine model revealed non-interference of the nail on the animal’s well-being confirming the safety and feasibility of the procedure. For the in vitro case, the strength of the restored bone was approximately one-third the strength of the normal (intact) bones, which suggested that improved bone strength could be achieved with further optimization in scaffold design and selection of materials. Another similar study examined the concept of percutaneous fracture fixation and the effect of scaffold reinforcement on fracture strength using porcine long bones (femora) in an ex vivo setting [12,52]. The study included two experimental groups and a control group; the controls were nonfractured (intact) femora and did not receive any intervention. The first experimental group received stents, wires, and bone cement; the second experimental group received bone cement only. Flexural stiffness, fracture energy absorption, and peak load at failure were evaluated for each of the groups. The physical parameters of the experimental groups were low compared to the control. Although there was no substantial difference between the two experimental groups (possibly due to the low sample size), a trend of higher stiffness, increased fracture resistance, and improved bending strength was noted in the group that received stents and Lunderquist extra stiff guidewires in addition to bone cement. These results advocate that the presence of reinforced material in the bone scaffolding site can further strengthen the mechanical properties of the fracture-repaired specimens. Table 3 provides a rapid overview of the state-of-the-art progress in the field of percutaneous osteoplasty.

## 3. Approaches to Improve Strength for Percutaneous Bone Interventions

### 3.1. Metallic Materials

**Figure 3:**
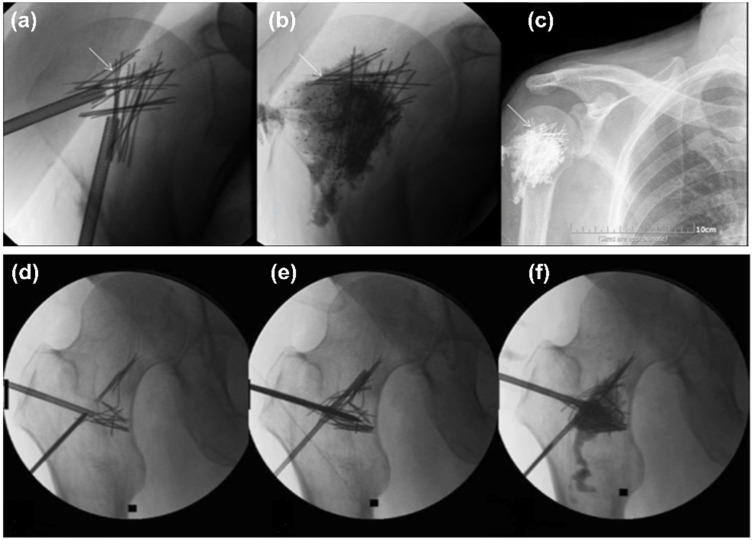
Percutaneous augmented osteoplasty with a metallic mesh containing 25–50 medical-grade stainless steel microneedles and PMMA cement [13,21]. Upper panel: A large, solitary, lytic metastatic lesion of the humeral head in a patient with esthesioneuroblastoma treated for pain palliation with augmented osteoplasty. (**a**) Under fluoroscopic guidance, 25–50 stainless steel microneedles (22-ga., 2–6 cm length) were inserted through the needle’s trochar, followed by, (**b**) PMMA cement injection, (**c**) X-ray image of the implant at 3-month follow-up showed needles in the original location (no migration). (Reprinted with permission, © 2015 Elsevier, [21]). Lower panel: Similar concept was applied to patients with multiple myeloma and painful lesion of the left femoral bone. (**d**,**e**) Insertion of metallic mesh (microneedles) through bone access needle in the lesion site in multiple orientations under fluoroscopic control, followed by, (**f**) PMMA cement injection. (Reprinted with permission, © 2016 Springer, [13]).

Scaffolds with material properties (e.g., stiffness) comparable to that of native bone provide better healing activity at the fractured site. These materials should ideally offer low deformations when acted upon by large forces, resulting in low strain and negligible motion (high stability) in the fractured area that encourages enhanced healing. PC with the use of these metallic materials has shown promise in patients with bone metastases. PMMA bone cement has high compressive strength making them ideal for spine applications; however, in the peripheral skeleton where other forces are dominant, e.g., bending, shear, tensile, torsion, etc., it may result in failure necessitating adjunct structural support [55]. To overcome this limitation, in one study, a metallic mesh containing 25 to 50 medical-grade stainless steel microneedles was inserted at the site of the metastatic lesion; this was followed by PMMA cement injection to create an overall “rebar” structure for repairing humeral head metastasis [21]. The patient had a reduced pain score after the procedure as well as moderate mobility. During the 3-month follow-up, the patient reported a significant drop in pain and improvement in mobility. In a later study, the same concept was applied to patients with femoral metastases; these patients also demonstrated an overall decrease in pain scores and improvements in mobility [13] (Figure 3). In another study, a flexible intramedullary nail and cementoplasty have been used to provide mechanical stability to long bones (tibia and femora) in cancerous patients [38]. These patients post-treatment demonstrated a significant decrease in pain and reduction in tumor volume (mean follow-up period: 16.17 ± 10.93 months (range 2–36 months)). The results of these studies further suggest that PC combined with additional reinforcement materials may provide improved stability and strength, and aid in tumor or defect containment in patients with metastasis or osteoporosis.

Similarly, Bensoussan et al. have used intralesional spindles to fortify the cement strength for palliation of malignant fractures of the humerus [56]. The reinforcement was necessary to overcome the poor resistance of PMMA cement to twisting and shear encountered by the humerus. The study was conducted on 6 patients that resulted in significant pain reduction (VAS score decreased from 10 to 1.5 six weeks after treatment) with minor procedure-related adverse events (4/6; 67%) relating to cement leakage (50%) and spindles migration from fracture site into areas of soft tissues (17%). No secondary fractures were reported during the follow-up period of 14.8 months [56].

**Table 3:** Summarized results of recent studies involving percutaneous osteoplasty with/without reinforcement for management of bone metastases.

Lesion Localization (Patients (n), (Year))	Intervention Type	Outcome	Complications	Reference
Hip and neck (*n* = 11, (2022))	Screw fixation and cementoplasty for pathologic bone fractures.	Significant decrease in pain score from 8.0 ± 2.7 to 1.6 ± 2.5, lower analgesic consumption from 70.9 ± 37 to 48.2 ± 46 mg/day, and improved EQ5D score from 42.5 ± 13.6 vs. 63.6 ± 10.3 (*p* < 0.05).	Minor subcutaneous hematoma (*n* = 1), and asymptomatic pulmonary cruciate embolism (*n* = 1).	[45]
Pelvic Ring (*n* = 50, (2021))	Percutaneous fixation with internal cemented screws (FICS) for prophylactic consolidation of large osteolytic tumors.	Postprocedural VAS: 0.76 ± 1.73 (preprocedural VAS: ≤3, out of 10). Long-term consolidation efficacy—98% (follow-up period 22 months).	Self-resolving hematoma (*n* = 2), inflammatory sciatic pain (*n* = 1), and focal pain at the ischial tuberosity (*n* = 1).	[46]
Hip, shoulder, chest, and jaw (*n* = 94, total 110 fractures, (2021))	Percutaneous image-guided screw fixation (PIGSF) of insufficiency, impending or pathological fractures.	Extremely low rates (<4%) of per-procedural (cement leak, induced fracture, or hematoma) and early complication (≤24 h) following PIGSF.	Delayed complications (>24 h, total: 18%) included infection (most frequent), focal pain, tumor seeding, screw loosening and fracture.	[47]
Periarticular load-bearing bones (*n* = 23, total lesion = 26, (2020))	Ablation, osteoplasty, reinforcement, and internal fixation (AORIF) of osteolytic lesions of the pelvis, hip, knee, and ankle.	Significant reduction in pain and function 2 weeks after procedure: VAS pain score decreased from 8.32 ± 1.70 to 2.36 ± 2.23, combined pain and functional ambulation score improved from 4.48 ± 2.84 to 7.28 ± 2.76, and Musculoskeletal Tumor Society score improved from 45% to 68%. No complications or infections noted from AORIF procedure during surgery or at 30 days.	None reported.	[57]
Femoral neck (*n* = 61, (2020))	FICS in metastatic patients with impending pathological fracture.	Short-term palliative efficacy: VAS score improved from 4.2 ± 3.2 to 1.8 ± 2.0 (*p* < 0.001) at 1 month after FICS. Long-term consolidation efficacy—92% (follow-up period >1.5 year).	Self-resolving hematomas (*n* = 3). Secondary fracture (5%).	[58]
Sternum (*n* = 9, (2019))	FICS for sternal fracture fixation or consolidation of osteolytic metastases.	Reduction in pain (Numeric Pain Rating Scale (NPRS) score: from 5.6/10 ± 2.8 to 1.1/10 ± 1.6) and decrease or withdrawal of analgesic consumption at post-procedural consultation. No secondary intervention required (follow-up period >1 year).	Hematoma (*n* = 1) and second pathologic fracture (*n* = 1). Secondary fracture (11%).	[29]
Pelvic bone (*n* = 126, total 178 lesions, (2019))	Percutaneous osteoplasty for treatment of pelvic bone metastases.	Pain score (VAS) decreased significantly post-procedure from 6.87 ± 1.33 to 3.33 ± 1.94 (day 3), 2.26 ± 1.59 (1 month), 1.89 ± 1.53 (3 months), 1.87 ± 1.46 (6 months), 1.90 ± 1.47 (9 months), and 1.49 ± 1.17 (12 months). Oswestry Disability Scores (ODI) scores changed significantly after the procedure and at each follow-up visit (3 days, 1-, 3-, 6-, 9-, and 12 months) compared to baseline.	Notable extraosseous cement leakage (28%) albeit without any clinical complication. No pain relief (6%) or pain aggravation (1%).	[25]
Spine (*n* = 69, total 102 spinal metastases, (2018))	Microwave ablation and cementoplasty for treatment of painful spinal metastases.	VAS score decreased from 7.0 ± 1.8 (preprocedural) to 2 ± 1.6 (2–4 weeks) and 2 ± 2.1 (20–24 weeks) postprocedurally. ODI score decreased from 46 ± 17.9 (preprocedural) to 24 ± 17.1 (2–4 weeks) and 24 ± 18.8 (20–24 weeks) postprocedurally.	S1 nerve thermal injury (*n* = 1) and skin burn (*n* = 1) (3%).	[59]
Pelvic bone and lower leg (*n* = 43, (2018))	Extraspinal cementoplasty for bone metastasis.	Improvement in pain score from 4.2 ± 3.6 (before cementoplasty) to 1.09 ± 2.4) (week 1) for 31 patients. Improvement in quality of life (48%) and disability (52%) at 22 months postprocedure (*n* = 21).	Cement leakage (12%), hematoma (2%), and acute respiratory distress due to infection (2%).	[60]

### 3.2. Regenerative Scaffolds

**Figure 4:**
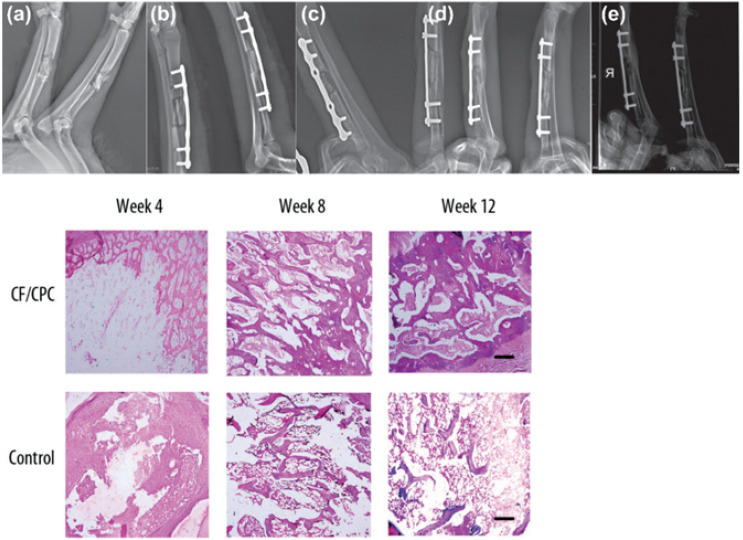
Chitosan fiber and calcium phosphate ceramics (CF/CPC) scaffold for fracture repair in weight-bearing long bones (radiuses) [61]. Upper panel: (**a**) X-ray images of both radii in adult dogs, right radius received CF/CPC scaffold, left radius were untreated (blank control). Radiographic images post-implantation at various time points in the experimental group: (**b**) 0 weeks, (**c**) 4 weeks, (**d**) 8 weeks, (**e**) 12 weeks. Lower panel: Histological examination of the bone-defect area tissue at 4-, 8-, and 12 weeks after surgery. The experimental group showed time-dependent slow resorption of cement and the formation of new bone tissues. In contrast, no biological activity occurred in the control group.

Regenerative scaffolds allow natural integration of injectates with native bone tissue, inhibit osteolytic activity, and promote bone cell proliferation. In one study, chitosan fiber and calcium phosphate ceramic (CF/CPC) scaffolds were examined for comminuted fracture repair of weight-bearing bones in a canine model [61]. Histological examination revealed that the fractures treated with the CF/CPC scaffold showed slow cement resorption and formation of new bone cells after week 4; by week 12, there was partial degradation of the scaffolding material (Figure 4). Mechanical testing demonstrated that bone with scaffolding had a failure strength 3 times stronger than the bone without scaffolding. This suggested that scaffolds can play an important role in bone remodeling and the treatment of fractures. 

The choice of bone cement for fracture repair has a significant impact on the strength and quality of the repair. Calcium phosphate or magnesium phosphate cement may be used as an alternative to acrylic (PMMA) bone cement and is being investigated for bone scaffolding and bone tissue engineering [62,63,64,65]. These scaffolds facilitate osteogenesis and osseointegration, which are suitable for bone healing and regeneration purposes. However, calcium phosphate cement has low mechanical strength and low resorption rate [66]; magnesium phosphate cement has a high exothermic setting reaction, inducing local thermal necrosis and the possible release of harmful ions [67]. Further research is needed to address these concerns.

**Figure 5:**
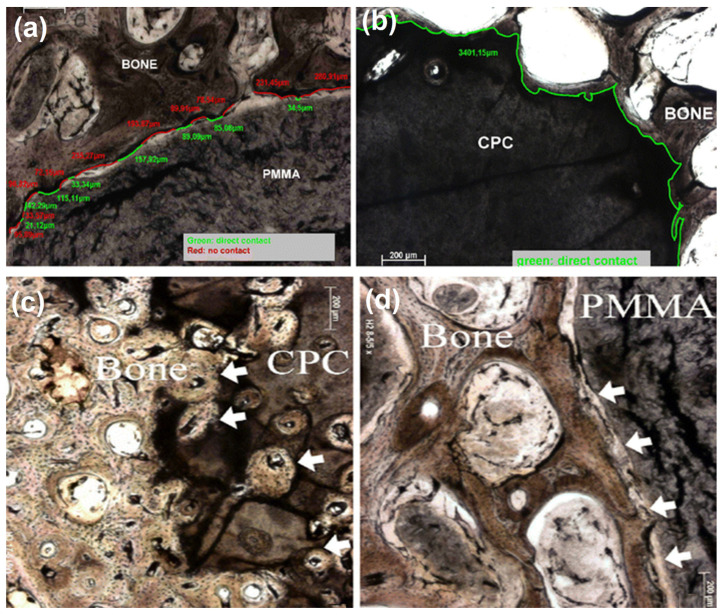
Osseous integration of calcium phosphate cement versus PMMA cement by histomorphometry in a canine model (osteopenic foxhound) that received intravertebral cement implant [63]. (**a**,**b**) PMMA implant (left) showing 30% of direct bone contact versus >80% of osseous integration with calcium phosphate cement (right) (imagery at 6 months; bone contact observed as early as 3 months after implantation) (green line—direct contact, red line—no contact). (**c**,**d**) Osteonal penetration occurred in the bone-calcium phosphate interface (left) but not in the bone-PMMA interface (right) (images after 12 months). Additionally, the study reported an increase in the number of osteons with time in calcium phosphate implants (not shown). (Reprinted with permission, © 2006 Springer, [63]).

Calcium phosphate cement has been used to treat fractures and metaphyseal defects. These types of cement are osteoconductive and seamlessly integrate with the bone tissues. In a clinical and pilot study, calcium phosphate cement was compared with PMMA cement in terms of the quality of fracture repair and osseointegration [63]. The clinical study, which assessed vertebral compression fractures, demonstrated significant cement resorption in the bone-cement interface with calcium phosphate cement versus PMMA cement. Similarly, the pilot study in a canine model showed significant ingrowth (>80%) of bone tissues and total bone coverage with calcium phosphate cement implants but only 30% bone contact with PMMA cement implants (Figure 5). Another study assessed the use of calcium phosphate cement versus autogenous iliac bone graft (the gold standard for filling metaphyseal defects) for tibial plateau fracture repair [62]. Calcium phosphate cement repair resulted in significantly higher fatigue strength and ultimate load compared to autogenous bone graft repair. The use of calcium phosphate cement for fracture repair in weight-bearing bones has also been assessed in a rabbit model [65]. In this study, a paste of tetra-calcium phosphate and dicalcium cement led to superior osseointegration and healing when compared to bone plates. Calcium phosphate cement demonstrates a promise for percutaneous applications, as these cement can easily integrate and degrade within the surrounding biological tissues in a timely fashion. 

### 3.3. Bone Morphogenetic Proteins

The delivery of bone morphogenetic proteins (BMPs) to the fractured sites using carrier materials such as natural or synthetic polymers, inorganic materials, or composite materials (listed in Table 4) favors tissue regeneration and remodeling resulting in improved healing response [68]. The availability of BMP in the scaffold allows migration, proliferation, and differentiation of regenerative cells in the vicinity of the injury. One study evaluated the efficacy of bone healing in a nonhuman primate fibular osteotomy model using human BMP-2 in various carrier matrixes [64]. The investigation found that BMP injected in calcium phosphate paste accelerated bone healing by approximately 40% compared to the healing of untreated osteotomy sites. With this combination, the mean torsion stiffness and maximum torque were equal to that of the intact fibula at 10 weeks versus torsion stiffness and maximum torque values of approximately 55% and 58%, respectively, for untreated osteotomy sites. Histological examination at this time point displayed bridging of the osteotomy sites with the bone for all carrier matrices. These results affirm that the incorporation and delivery of various biological factors to the compromised site significantly alters the healing and regeneration response and improves the outcome of percutaneous interventional strategies.

## 4. Mechanical Characterization of Bone/Bone Implant Devices

Mechanical characterization of the bone interventional device is necessary to examine its use and fit for a potential clinical application. Because of the bioengineering aspects involved in designing and executing these tests, it can be overwhelming to decide on relevant mechanical tests needed for proof-of-concept evaluation. Herein, we provide a brief overview of the common tests that are essential to answer basic questions related to fracture strength, durability, and applicability; comprehensive test methods have been reviewed elsewhere [69,70,71,72,73,74]. Successful execution of these tests provides insights into whether revisions are needed for the current innovation or if the device is technically fit to advance into the next step, for instance, evaluation in animal models. When the device fails to pass an acceptable strength test during mechanical characterization, a revision is necessary, and this feedback is obtained early in the developmental pipeline. The common extrinsic parameters of interest calculated from the force-displacement curve are yield load (N), ultimate/ breaking load (N), stiffness (N/mm), deformation (mm), and fracture energy (N.mm or mJ). These parameters inform the maximum force that the product can withstand before failing. Similarly, the intrinsic parameters of interest obtained from the stress–strain curve include yield stress (Pa or N/m^2^), ultimate /breaking stress (Pa), strain (mm/mm) modulus of elasticity (Pa), fracture toughness (N∙m^−3/2^, Pa∙m^−1/2^), and toughness (J∙m^−3^). These quantities provide information on the material property and behavior and help judge their fit for a particular clinical application.

### 4.1. Flexural Test

The bone in vivo is subjected to multiple forces from daily muscular activity, impact, and gravity that causes bending, torsion, extension, and compression. Because of the natural curvature of the long bone, bone bending is the most common phenomenon induced in vivo when the bone is subjected to these internal loads. To evaluate the bending properties of the bone, one can either choose a 3-point or a 4-point test. With a 3-point test configuration, a shorter gauge specimen can be conveniently examined, whereas, a 4-point test requires a relatively longer gauge specimen. In contrast, the 4-point test has the advantage of simulating a pure bending phenomenon with minimal shear effects (shear: force acting parallel to the material’s cross-section to produce a sliding failure). In the 3-point bend test, there is an inherent influence of shear, which affects the assumptions and outcomes of these tests, e.g., increased deflection/strain or early arrival to failure from low intensity applied force/stress. However, these effects can be reduced if the experiment is designed judiciously. For instance, for a 3-point flexural test, the ASTM D790 standard recommends using a span-to-thickness (s-t) ratio of 16:1 to reduce the shear effects [75]. As per this ratio, the length of the beam (specimen) requires to be longer than its vertical depth or diameter (in the case of circular cross-section). An s-t ratio below 14:1 is not recommended because of the high apparent flexural strength from shearing. For highly anisotropic composites, the ratio may be increased to 20:1, 32:1, 40:1, or 60:1 to minimize the shear effects [75,76,77]. 

During mechanical testing, a preconditioning stage precedes the main loading stage where low loads are cyclically applied to ensure loading fixtures are in direct contact with the bone surface. This helps to overcome geometrical irregularities common at the bone-fixture interface, which may otherwise lead to specimen instability on the fixture when loads are applied. Whole bone testing using a bend test can provide accurate measurements of its extrinsic properties but the measurement of intrinsic material properties may not be accurate due to geometric irregularities of the specimen and the assumptions involved. A compression or tensional test using a small-sized (cut-out) specimen is recommended in such situations.

#### 4.1.1. Three-Point Bending Test

**Figure 6:**
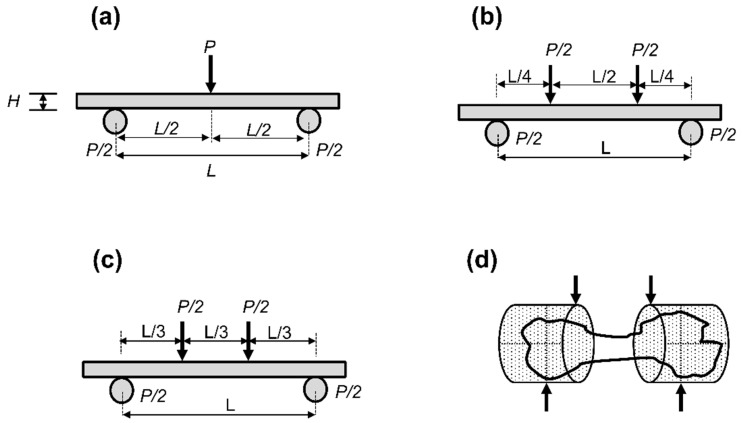
Flexural test setup. (**a**) 3-point test, (**b**) quarter-point loading (4-point test), (**c**) third-point loading (4-point test), (**d**) specimen potted for a 4-point test (to overcome its short gauge length). Specimens can be potted in circular or rectangular cups. *p*–applied load, *L*–gauge length (distance between supports), *H*–specimen vertical depth (thickness).

In a 3-point bend test configuration, the specimen is placed over the fixture, supported at 2 outer points, and a load is applied vertically at the center of the specimen (Figure 6a). The test setup is simple and more commonly used to test the bones of small animals because of the limitations of the gauge length; however, it remains a popular method for testing bones of large animals as well. In [43,44], a three-point bend test has been used to evaluate the bending power (force applied, (N)) and bending strength (ratio of bending power to the elliptical cross-sectional area of the specimen, (N/mm^2^)) of fixation imparted by insertion of bone cement and/or filler materials and compared with intact specimens. In 3 point test, there is an unequal stress concentration at the center of the specimen, hence, pure bending is difficult to obtain. The bending moment and shear force are maxima in the center, while at all other locations, shear is constant, and the bending moment declines from the center to the edges. To achieve uniform bending over the entire specimen length, 4-point bending needs to be considered.

A three-point bending test has been applied to evaluate the biomechanical strength of osteoplasty with or without Kirschner wire augmentation in a cadaveric human (long bone) diaphysis model [48]. The study was conducted in three groups (*n* = 30, total): No consolidation (group 1, *n * =  10), osteoplasty alone (group 2, *n * =  10), or K-wires augmented osteoplasty (group 3, *n * =  10). Mechanical parameters such as load to fracture (N), and Young’s modulus (N/m^2^) were quantified. The typical values (mean ±SD) of load to fracture and Young’s moduli were 1078 ± 370 N and 397 ± 140 N/m^2^ (group 1), 1222 ± 338 N and 445 ± 153 N/m^2^ (group 2), and 1230 ± 293 N and 431 ± 140 N/m^2^ (group 3), respectively [48]. 

#### 4.1.2. Four-Point Bending Test

The 4-point bend test involves placing the specimen over the fixture, supported at 2 outer points (bottom), and loading the specimen at 2 inner points (top) (Figure 6b,c). In this test setup, the specimen under test is in direct contact with 4 forces (2 loading forces on top and 2 supporting reaction forces on its bottom). The loading configuration for this test can be of two types: quarter-point loading (Figure 6b) or third-point loading (Figure 6c). In quarter-point loading, the distance between the inner loads is one-half of the total distance between the supports, and the distance between the support and its nearest inner load is one-quarter of the distance between the supports. In third-point loading, the distance between the inner loads or the distance between the outer support and the nearest inner loads is the same and is equal to one-third of the distance between the supports. The 4-point bending test theoretically ensures pure bending and the absence of shear forces between 2 loading points. In [12], a four-point bend test has been used to evaluate bone mechanical properties such as peak load at failure (force applied, (N)), flexural stiffness (bending moment applied to angular deflection, (N∙m/θ)), and work done to fracture (area under the force-deflection curve, (N∙mm)), to determine the quality of fixation from the use of filler materials. 

A general test scheme using a 3-point and a 4-point configuration has been depicted (Figure 6). Table 5 summarizes the mechanical parameters that can be obtained from 3-point and 4-point loading configurations [75,76,77,78,79].

### 4.2. Potting Bone Ends to Comply with Four-Point Test and Multidirectional Testing

It can be difficult to evaluate the bone specimen of a small animal in a 4-point bend test configuration because of the short gauge length. When such specimens are subjected to a 4-point bend test, the distance between the internal loading pins tends to be very small, leading to a setup similar to that of a 3-point bend test. Hence, to overcome the limitation, the ends of the bone can be potted in cylindrical or square cups filled with a low melting point bismuth alloy (Wood’s metal/Cerrobend) or bone/dental cement. Subsequently, the loads can be applied directly over the potted surface (Figure 5d) [80,81]. This method also helps to securely anchor irregular specimens over the 4-point fixture during testing. As a potential downside, improperly aligned potted ends can introduce inadvertent shear effects but can be managed with custom-designed alignment fixtures, as discussed in [82,83,84]. 

**Figure 7:**
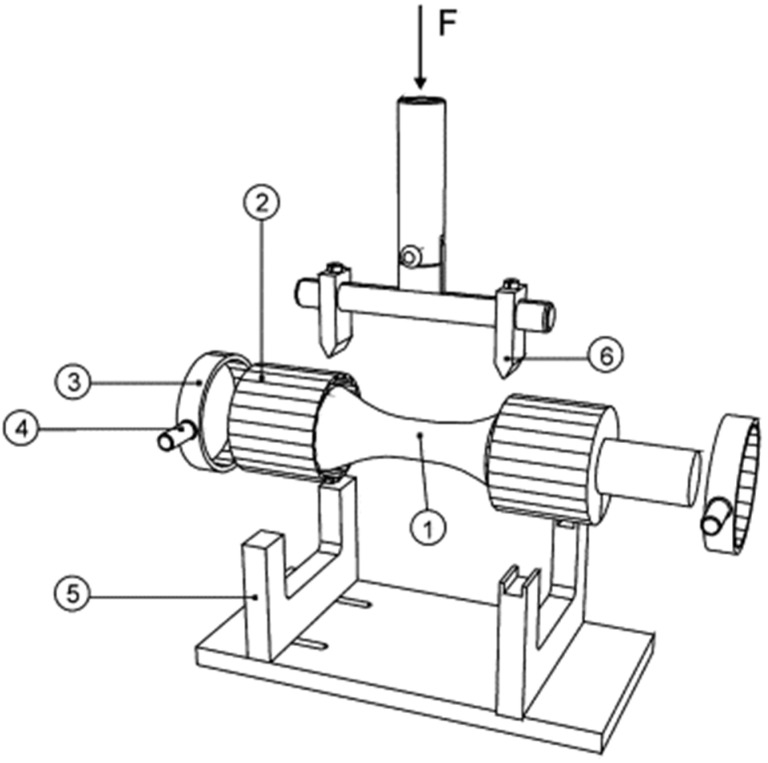
Custom-designed 4-point bend test fixture to overcome bone anisotropy [80]. The setup evaluated bone bending stiffness in 24 planes (360°) upon application of a non-destructive force (no plastic deformation). The specimen was simply supported at its end and not rigidly fixed. (**1**) Test specimen, (**2**) grooved metal cups, (**3**) corresponding rings, (**4**) lug for holding the specimen in the fixture, (**5**) 4-point supporting fixture (bottom), and (**6**) 4-point loading fixture (top). (Reprinted with permission, © 1998 Elsevier, [80]).

The bone’s unique geometry and material anisotropy make its bending properties dependent on the testing plane. It may, therefore, be necessary to perform flexural testing in several directions to accurately quantify its mechanical properties. Bramer et al. developed an optimized mechanical testing model to characterize bone properties for use with 4-point testing (Figure 7) [80]. This test configuration was modified from the test setup described by Foux et al., where the authors used a 3-point testing scheme in 24 directions, perpendicular to the long axis of the bone, to characterize its mechanical properties [81]. In the study by Bramer et al., the test specimens were fitted in cylindrical metal cups filled with low melting bismuth alloy [80]. The metal cups had 24 grooves corresponding to 24 testing orientations. The specimen was kept in a custom fixture and subjected to nondestructive testing under axial loading in a 4-point bend configuration. During the test, the specimen was retrieved from the fixture, rotated 15°, and replaced in the fixture for testing in the succeeding orientation. This procedure was carried out until testing was completed in 24 directions (360°), i.e., throughout the specimen’s circumference. The mechanical properties were then characterized in terms of stiffness index, area ratio, flatness ratio, and inclination for these orientations.

### 4.3. Torsional Test

Since long bones in the body are continuously subjected to twisting forces, it is important to evaluate the mechanical performance of the bone or bone-implant devices under torsion [85]. Torsional testing applies loading to the entire specimen’s length to simulate fractures commonly encountered in clinics. In contrast, compression or flexural test applies concentrated load that may lead to local deformation and the appearance of late fracture or specimen crushing. A torsional test is conducted to obtain useful information such as torsional shear stress or strain, maximum torque, shear modulus, etc.

Quinnan et al. have used torsional testing to compare the mechanical performance imparted by the PMMA cement-coated intramedullary nails for the fixation of tibial diaphyseal fractures [86]. The cement containing antibiotics is generally applied to the nail to prevent or fight against potential infection. Eight tibial bone phantoms were used that underwent simple transverse fracture of the tibia (2 mm gap) and fixed with PMMA-coated or uncoated (regular) titanium 8 mm DePuy ACE nails. For the torque testing, the relative range of motion (RoM) was captured between proximal and distal bone fragments upon twisting (±0.5 to ±3.0 N·m at 0.25 Hz) using a video-camera system in sagittal, coronal, and axial planes. No significant difference in the RoMs of the two constructs was found indicating cementing does not affect or offer additional mechanical rigidity to the nails [86]. 

### 4.4. Hardness/Indentation Test

The bone is a composite structure that obtains unique biomechanical properties from the spatial organization of inorganic (hydroxyapatite crystallites, ~60%) and organic (mostly, type I fibrillar collagen, ~30%) material in a heterogeneous matrix [87]. The hierarchical molecular organization of constituent elements at a particular site determines the biological/mechanical properties of the bone (bone quality, fragility, load bearing capacity, etc.), and varies throughout the bone geometry. It is, therefore, important to ascertain the properties of the constituents elements at the micro/nano level in various regions to understand the structural performance of the bone. Bone hardness testing examines the ability to resist deformation when penetrated with an indenter [88]. Hardness testing is classified based on the size of the indenter (Brinell, Rockwell, Vickers, Knoop) employed for testing and the hardness value typically varies according to the sectional region that is indented [89]. Hardness testing provides a better understating of the strength of the bone (bone quality) in an in vivo environment and is particularly useful for bone-related research.

Ibrahim et al. have used a Vickers tester to compare microhardness at different regions of the tibial shaft treated without/with boiled water (100 °C for 30 min) or sodium hypochlorite (NaOCl) solution [90]. The specimens were loaded with a 50 g load and a 10 s dwell time with a minimum distance of 3d (d, diagonal of indentation) between any two consecutive indentations. The study reported a substantial decrease in bone hardness with both treatments (25% less with hot water and 58% less with chemical at 2 h) from thermal denaturation of collagen (hot water) and chemical degradation of the organic and inorganic matrix (NaOCl treatment) [90]. The technique applied in this study can be adapted to study the fundamental variations in microhardness between a normal bone and a metastatic or osteoporotic bone to identify a qualified implant based on its mechanical competence.

### 4.5. In Silico Test

In silico testing are experiments conducted virtually using mathematical modeling software(s) typically in an advanced computing environment. In the software, the bone computer model and input conditions along with constraints are fed and output in terms of, for instance, reactionary forces and displacements are predicted based on various models [91]. In silico test is being chosen over traditional in vivo or ex vivo assays as it significantly downsizes the time, cost, and manpower involved in specimen procurement, preparation, and experimentation, and eliminates the need for dedicated test equipment [92]. In addition, virtual experiments overcome ethical and regulatory considerations. On the other hand, the accuracy of prediction is dependent on the quality of the input data, thus, requiring an experienced user to model and execute these tests. A typical workflow of finite element analysis for bone testing involves the acquisition of a bone model with/without defects (via imaging a real specimen with a high-resolution scanner [micro-CT, ultrasound] or using an off-the-shelf model), segmentation, 3D model generation, and mesh optimization, application of load and boundary conditions, and finite element analysis for output calculation [93]. Typical output parameters include maximum load, stress distribution patterns (von Mises), deformation, stiffness, etc. 

**Figure 8:**
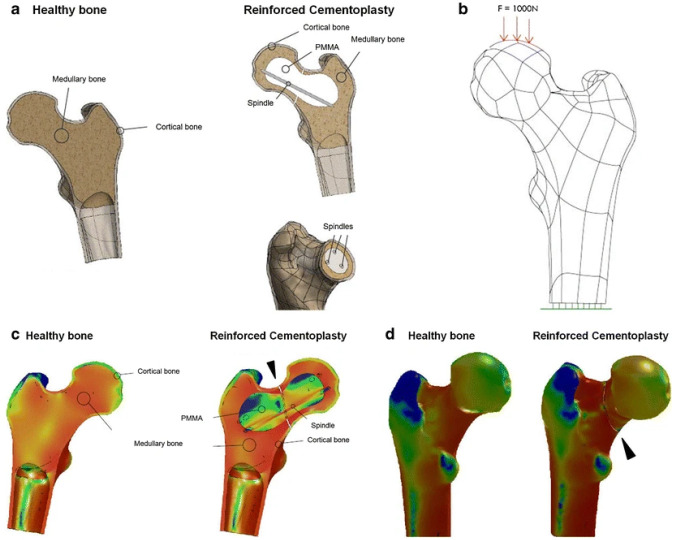
In silico testing of reinforced cementoplasty of the proximal femur [94]. (**a**) Normal healthy bone (left) and a diseased bone (right) model that received cementoplasty plus spindles, (**b**) proximal femur loading (1000 N) to simulate physiological loading conditions, color-coded depiction of forces (**c**) within, and on the (**d**) surface of cervicotrochanteric region (red—high magnitude forces, blue—low magnitude forces). A fracture line was included in the diseased bone to model the femoral neck fracture (shown by black arrowhead). No significant difference in mechanical constraints was observed between the normal bone and RC-repaired bone. The simulations were executed in Solidworks software (Dassault Systèmes, France). (Reprinted with permission, © 2017 Springer, [94]).

Cementoplasty alone provides poor stability with a risk of secondary fracture. To improve bone stability in patients with unstable malignant lesions of the cervicotrochanteric region, Premat et al. have proposed reinforced cementoplasty (RC, cementoplasty plus internal fixation using dedicated spindles) as a therapeutic model for patients deemed unsuitable for open surgery [94]. A simplified computational model (designed in Solidworks software) of the proximal femur was used to evaluate the mechanical strength imparted by the fixation (Figure 8). The diseased model was treated by RC, included a femoral neck fracture, and was examined by applying 1000 Newton compression force on its head. The study reported that in the areas of intervention, mechanical constraints were similar to that of a normal (intact) bone with the parameters far-off from failure conditions [94]. This indicated that RC fixation was a viable approach to improving biomechanical resistance to fracture.

## 5. Summary and Future Direction

Bone is the “hot spot” for the spread of cancer from commonly affected primary organ sites such as breast, kidney, lung, or prostrate that principally leads to painful lytic bone defects and/or predisposition to fracture [95]. Up to 20–40% of cancer patients have uncontrolled bone metastases and skeletal-related events representing a significant patient population who may need an immediate bone intervention in the future [23]. Percutaneous osteoplasty, a procedure similar to vertebroplasty or kyphoplasty in the spine, is an effective radiological procedure used widely for the treatment of benign or malignant (extraspinal) bone lesions [18]. It involves the injection of bone cement into the painful osteolytic lesions via catheters and guidewires under digital fluoroscopic guidance. Percutaneous cementoplasty has been reported to be a safe procedure without major complications, immediate or delayed [13,33,36,45]. It stabilizes and strengthens the bone matrix by forming a cohesive bond unifying bone elements that prevent microfractures and result in pain regression. 

Percutaneous osteoplasty with reinforcement is emerging as a new therapeutic model for patients with induced or impending fractures from bone metastases or osteoporosis. These patients often present with compromised bone strength or weak health (strictly “non-surgical” patients) that prevents them from undergoing invasive surgical fixation procedures under general anesthesia. The supplementation is required as cement augmentation alone lacks sufficient strength for bone union and stabilization, esp. weight-bearing bones. Due to an obvious lack of qualified materials for reinforcement-osteoplasty, researchers have improvised radio-surgical use materials for proof-of-concept verification with limited success [12,13,48]. Reinforcement osteoplasty involves strengthening bone toughness with durability-awarding materials that mimic “rebar” in the background of cementing material. Because of the percutaneous nature of the application, materials need to be sized accordingly so that they can fit and be delivered via a small opening or “access” made through the skin into the cortical region. The requirements of reinforcement implants can be a major design/ technical challenge as it requires them to be biocompatible, flexible (to allow insertion at an angle), and miniature as well as sturdy (after deployment at the target site) to render resilient support. Failure to fulfill these criteria may produce no appreciable results. For instance, authors reported no added benefit with osteoplasty (PMMA cement) alone or in combination with Kirschner wires (K-wires) to resist bending stress in a cadaveric human diaphyseal model possibly from the use of suboptimal composite materials [48]. The volume of cement injected also plays a key role in determining the strength of fixation. Because of the enormous built-in back pressure and the quick onset of polymerization, it may not be usually feasible to manually inject cement volumes greater than 6–8 mL in the form of a single cohesive ball. In such scenarios, the use of an automated hydraulic-force cement injector [96,97] or a robotic injection device as described by Garnon et al. [98] may be preferred.

In vitro mechanical assessment provides insights into the feasibility of these novel procedures and devices that are designed to undertake loads and provide stability for a wide array of orthopedic applications. Various test methods have been developed to characterize the mechanical properties of these devices, and the intended use and location in vivo determine the types of tests that need to be executed and the parameters to look for. PMMA bone cement has superior axial compressive strength and capacity to withstand compression in flat bones like the spine and hip [40]. However, it has low torsional, shear, and bending stress handling capacity [36] and carries a risk of secondary fracture when applied to overcome long bone neoplastic defects. Studies report 8–9% of secondary long bone fractures in metastatic patients following osteoplasty [30]. In the event of secondary fracture, further fixation is almost impractical because of the permanent closure of the internal void from cement filling. Calcium phosphate cement may be preferable over PMMA bone cement for fracture repair in the given context because of its superior biological and osteogenic properties [99]. However, the inferior mechanical strength of calcium phosphate cement over PMMA cement requires further research to address this limitation.

Percutaneous osteoplasty with a range of adjunctive reinforcement is implemented on a case-by-case basis in clinics for the consolidation of long bone fractures (impending/pathological) and has produced encouraging results [13,37]. However, these studies lack adequate preclinical biomechanical characterization and the outcomes may be limited to short-term gains. Further studies are needed to evaluate the long-term benefits of these procedures. Lack of osteointegration with the native tissues and non-osteoconductive properties of the implant are the most common causes of infections and imminent failures; therefore, research efforts need to be directed to find optimal solutions with a focus on physical, mechanical, and biochemical factors present in vivo. The challenges can be partly addressed with the development of next-generation bone cement composed of various osteogenic growth factors and possibly antitumor/anti-inflammatory drugs that can positively impact molecular and cellular processes and allow bonding and integration with the skeletal tissues in the targeted region without inflammation. The transformation of bone cement into an osteosynthetic material instead of being limited to a space filler heralds new bone growth and may alleviate concerns related to poor stress handling capability. Extraosseous cement leakage during percutaneous osteoplasty procedures is also a common concern that can potentially cause inflammation, pain, and tissue injury, and can be overcome with the use of an optimally viscous cement and following a cautious surgical approach [100]. While there is a clear lack of sufficient clinical studies to support percutaneous reinforcement, the potential benefits in terms of pain relief, mechanical stability, and early facilitation of weight-bearing bones with bioengineered scaffold should not be discounted. Proper guidance on patient selection, surgical efficacy, and related complications based on the outcomes of large-scale studies and longer follow-ups are awaited. Moving forward, emphasis needs to be given to combination treatment that includes novel biomaterials with biocompatible and bioactive properties that can provide synergy in supplementing bone strength by aiding new bone formation, restoring anatomical defects and physiological function, and pain regression for percutaneous applications.

## Figures and Tables

**Table 4:** Common types of carrier materials for delivery of bone morphogenetic proteins.

Type	Examples	Advantages	Disadvantages	Ref.
Natural	Collagen, Hyaluronans, fibrin, chitosan, silk	Biocompatible, Bioresorbable	Procurement, disease transmission, immunogenicity	[68]
Synthetic	Polylactide, polyglycolide, PLGA	Design flexibility, no disease transmission	Inflammatory response, poor clearance due to high molecular weight	
Inorganic	Calcium orthophosphates, Bioglass	Biocompatible, Bioresorbable, osteoconductive	Low mechanical property, lack of macroporosity for cell infiltration	
Composite	Composite materials that include natural, synthetic, and/or inorganic components	Biocompatibility, improved handling	Phase separation	

**Table 5:** Mechanical parameters quantifiable from a flexural test (3-point and 4-point testing).

Parameter	Center Point Load (3-Point Test)	4-Point Test		Reference
		**Third-Point Load**	**Quarter-Point Load**	
Flexural strength (Bending stress)	$\frac{PLH}{8I}$	$\frac{PLH}{12I}$	$\frac{PLH}{16I}$	[75,76,77,78,79]
Flexural modulus	$\frac{PL^{3}}{48\delta I}$	$\frac{PL^{3}}{56.5\delta I}$	$\frac{PL^{3}}{70\delta I}$	
Deflection	$\frac{PL^{3}}{48EI}$	$\frac{PL^{3}}{56.5EI}$	$\frac{PL^{3}}{70EI}$	
Bending moment	$\frac{PL}{4}$	$\frac{PL}{6}$	$\frac{PL}{8}$	

*p* = applied load, *L* = gauge length, *H* = thickness (depth) of the specimen, *I* = area moment of inertia, δ = deformation, *E* = Modulus of elasticity.

## Data Availability

Not applicable.
